# Supplementary material for: DMRforPairs: identifying Differentially Methylated Regions between unique samples using array based methylation profiles
Source: BMC Bioinformatics. 2014 May 15;15:141. doi: 10.1186/1471-2105-15-141 (PMC4046028; doi:10.1186/1471-2105-15-141)
Supplement: Additional file 2 — DMRforPairs output for the comparison of A431-MCF7 and NA17018-NA17105. Please start from the HTML files in each folder. Available via the BMC Bioinformatics website. [file 1471-2105-15-141-S2.zip › 1394847754114233_MOESM2_ESM/A431_MCF7/figures/6511.pdf]

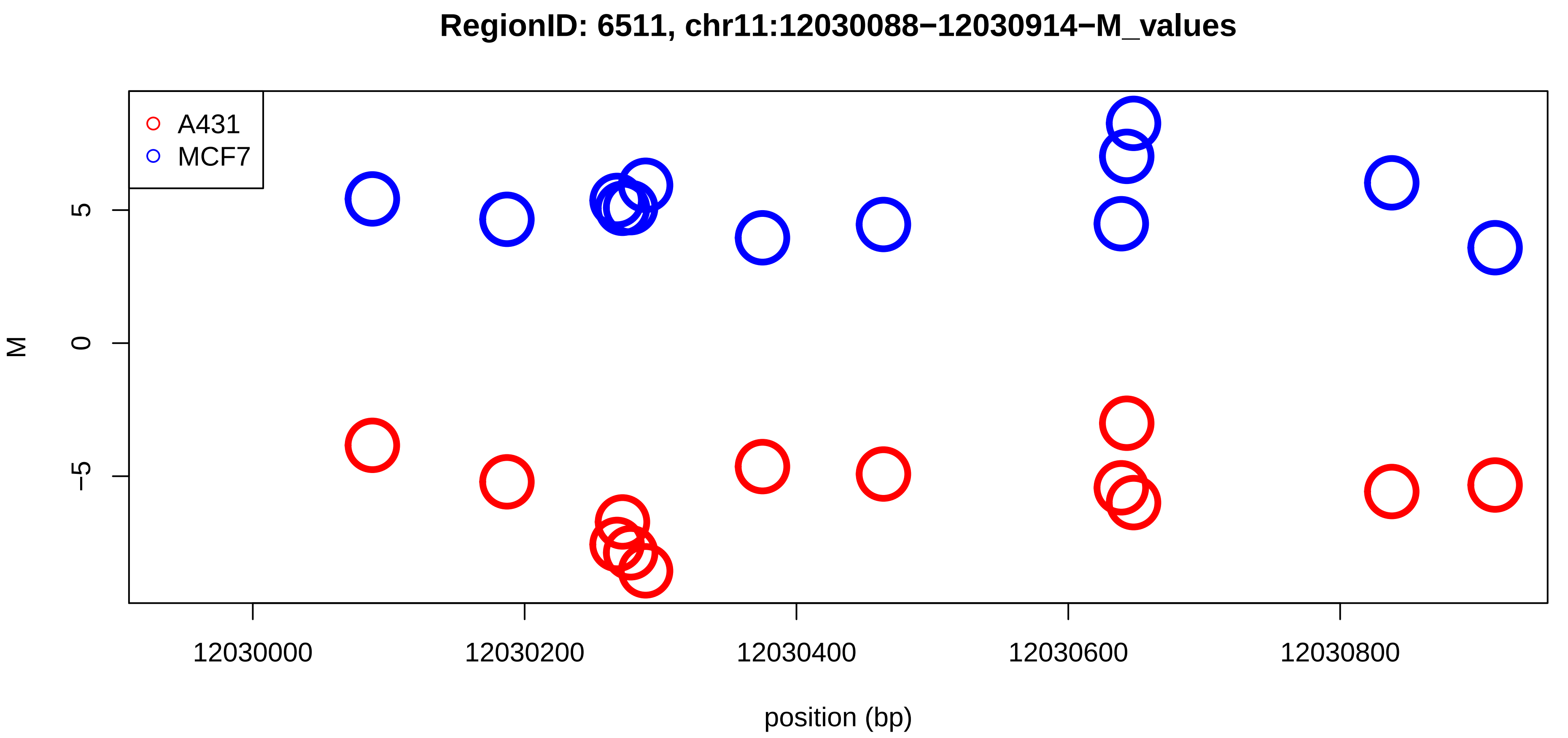
RegionID: 6511, chr11:12030088−12030914−M_values
A431
MCF7
M
5
0
−5
12030000
12030200
12030400
12030600
12030800
position (bp)

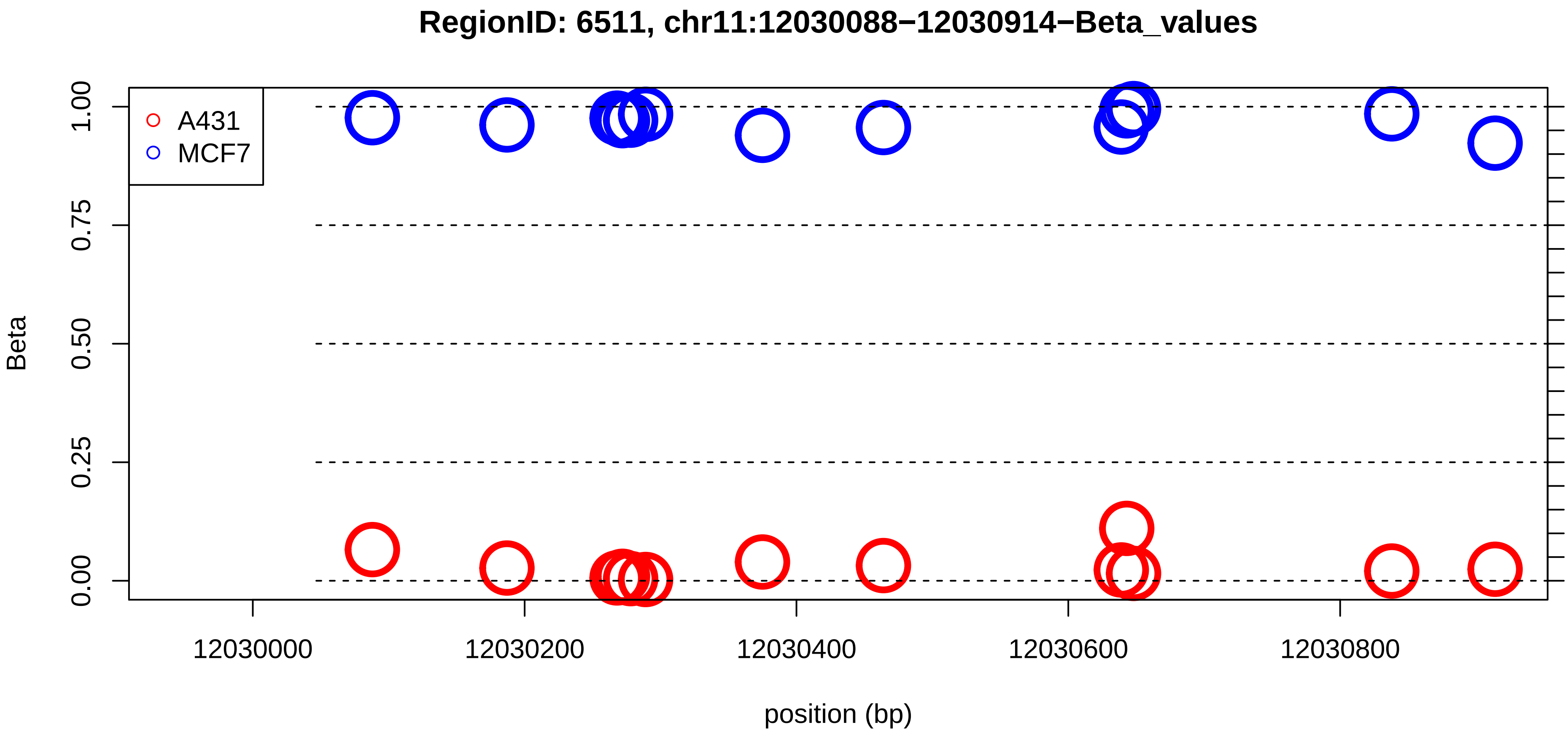
RegionID: 6511, chr11:12030088−12030914−Beta_values
A431
MCF7
Beta
1.00
0.75
0.50
0.25
0.00
12030000
12030200
12030400
12030600
12030800
position (bp)

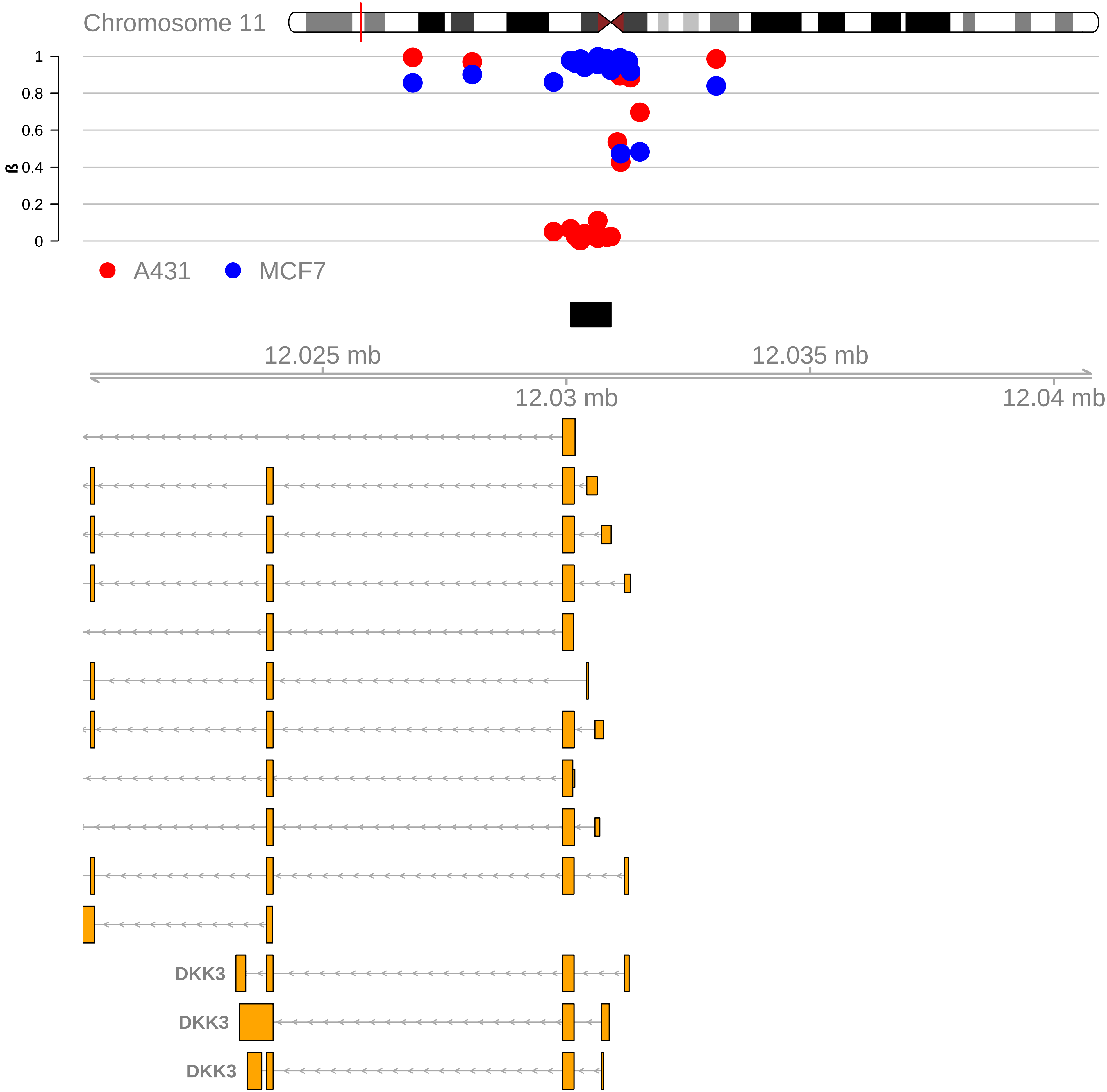

Chromosome 11
1
0.8
0.6
ß 0.4
0.2
0
A431
MCF7
12.025 mb
12.035 mb
12.03 mb
12.04 mb
DKK3
DKK3
DKK3
